# Effect of low dose, short-term creatine supplementation on muscle power output in elite youth soccer players

**DOI:** 10.1186/s12970-017-0162-2

**Published:** 2017-02-07

**Authors:** Aquiles Yáñez-Silva, Cosme F. Buzzachera, Ivan Da C. Piçarro, Renata S. B. Januario, Luis H. B. Ferreira, Steven R. McAnulty, Alan C. Utter, Tacito P. Souza-Junior

**Affiliations:** 1 Dirección de Investigación, Universidad Mayor Santiago de Chile; Universidad Santo Tomás, Talca. Chile. Carrera de Educación Física, Santiago de Chile, Chile; 2 Department of Physical Education, North University of Parana, Londrina, Brazil; 3 School of Physical Education, Max Planck Faculty, Indaiatuba, Brazil; 4 Department of Physical Education, Research Group on Metabolism, Nutrition and Strength Training, Curitiba, Brazil; 5 Department of Health and Exercise Science, Appalachian State University, Boone, USA

**Keywords:** Creatine supplementation, Wingate test, Anaerobic performance

## Abstract

**Background:**

To determine the effects of a low dose, short-term Creatine monohydrate (Cr) supplementation (0.03 g.kg.d^−1^ during 14 d) on muscle power output in elite youth soccer players.

**Methods:**

Using a two-group matched, double blind, placebo-controlled design, nineteen male soccer players (mean age = 17.0 ± 0.5 years) were randomly assigned to either Cr (*N* = 9) or placebo (*N* = 10) group. Before and after supplementation, participants performed a 30s Wingate Anaerobic Test (WAnT) to assess peak power output (PPO), mean power output (MPO), fatigue index (FI), and total work.

**Results:**

There were significant increases in both PPO and MPO after the Cr supplementation period (*P* ≤ 0.05) but not the placebo period. There were also significant increases in total work, but not FI, after the Cr supplementation and placebo periods (*P* ≤ 0.05). Notably, there were differences in total work between the Cr and placebo groups after (*P* ≤ 0.05) but not before the 14 d supplementation period.

**Conclusion:**

There is substantial evidence to indicate that a low-dose, short-term oral Cr supplementation beneficially affected muscle power output in elite youth soccer players.

## Background

The physiological demands of soccer require a well-developed aerobic and anaerobic fitness of the players [1]. Although the relevance of aerobic fitness levels in soccer has been confirmed by previous studies [2, 3], it is currently recognized that the most decisive actions during a soccer game are covered by anaerobic metabolism [4]. Indeed, anaerobic energy release plays a key role on the performance of a number of relevant activities during a soccer match, such as sprinting, jumping, tackling, kicking, and changes of direction [5], which may determine the final outcome of a game.

Previous research has shown that maximal or near maximal exercise bouts require a high skeletal muscle adenosine triphosphate (ATP) turnover rate [6]. As intramuscular ATP storage is able to sustain muscular activity for only few seconds, ATP must continually be resynthesized for activity to continue. Gaitanos et al. [6] have reported that the majority of the energy required to resynthesize ATP during short-term, maximal exercise bouts is primarily provided by a combination of phosphocreatine (PCr) degradation and anaerobic glycolysis. However, when PCr becomes depleted, performance deteriorates because ATP cannot be resynthesized at the rate required. That perspective has led some researchers to suggest that interventions, which increase resting levels of PCr availability, might delay PCr depletion and attenuate the decline in ATP provision during maximal or near maximal exercise bout [7]. Moreover, it has been suggested that higher level of PCr availability might accelerate the rate of PCr resynthesis after multiple bouts of intense exercise [7, 8].

A popular dietary strategy for increasing resting levels of PCr availability and/or maximizing the team sports players’ capacity to perform high intensity, exercise bouts is supplementation with oral Creatine monohydrate (Cr). Indeed, a number of studies confirmed that short-term, Cr supplementation (20–30 g^.^d^−1^ during 3–6 days), in amounts substantially in excess of the normal dietary intake, can elevate the total muscle Cr content (PCr + Cr) by approximately 20%, one third of which is in the form of PCr [7, 9, 10]. However, the results of studies investigating the effects of short-term, Cr supplementation on maximal exercise performance have been equivocal. Several studies have demonstrated an improved high intensity exercise performance after short-term, Cr supplementation [7, 8, 11, 12], whereas several others have reported no beneficial effect [13, 14]. Some of these conflicting results are likely to be associated with differences in the Cr dosing regimens for the duration of the investigation. Whereas the majority of the studies used a typical Cr supplementation regimen including a “loading phase” of about 20 to 25 g.d^−1^ (0.3 g.kg.d^−1^) for 5 to 7 days to maximize muscle total Cr content, followed by a “maintenance phase” of about 2 to 5 g.d^−1^ [15], more recent studies have used alternative Cr supplementation regimens with doses of Cr as low as 2 to 5 g.d^−1^ (0.03 g.kg.d^−1^) for a longer period of 2 to 6 weeks without using a prior “loading phase” [16–18]. Importantly, both Cr dosing regimens can increase total muscle Cr content and promote ergogenic effects. A recent study presented by Rawson et al. [12] has shown that ingesting low doses of Cr as low as ~2.3 g.d^−1^ (0.03 g.kg.d^−1^) for 6 weeks significantly increased the plasma Cr levels and enhanced the resistance to fatigue during multiple and high intensity exercise bouts. Although this study has found the lowest effective dose of Cr seen in the current literature, it is clear the duration of the Cr supplementation period was extended beyond the usual duration of typical Cr supplementation regimens [12, 18]. To the authors’ knowledge, the minimum duration of supplementation with Cr that is required to promote ergogenic effects on performance when ingesting doses of Cr as low as 2 to 5 g.d^−1^ (0.03 g.kg.d^−1^) has not been clearly defined. However, there is a shortage of scientific data concerning the possible effects of oral Cr monohydrate supplementation on specific performance in sports such as soccer, which consists of intermittent repeated bouts of high-intensity exercise. The effect of Cr supplementation on young competitive athletes has received much less research attention. Therefore, the purpose of this study was to examine whether a low dose of 0.03 g.kg.d^−1^ of Cr supplementation for a short duration (only 14 days) affects muscle power output in a group of elite youth soccer players.

## Methods

### Subjects

Nineteen elite youth soccer players volunteered to participate in the study, which was performed in accordance with the Helsinki Declaration of 1975. The research was approved by the Ethics Committee of the Federal University of São Paulo. Each participant and parent gave their written informed consent after explanation of the study purpose, experimental procedures, possible risks and benefits. All the volunteers signed a free and informed consent term before participation. The selected players were members of the same team, played in national and international championships at the time of the investigation, and had a mean of 6.9 ± 3.9 years. of continuous soccer training and competition background. During the months before the beginning of the experimental period, players trained five times a week (~90 to 120 min per session), with an official soccer match taking place at the end of the week. Each training session consisted mainly of technical and tactical skill development (60% of the training time) and physical conditioning with emphasis on anaerobic and aerobic performance development. During the experimental period, players trained only two times a week and no official soccer match was played.

Experimental procedures were conducted in the middle stage of the competitive season (March to October), in the weeks where no official soccer matches were played. All players were free from current injuries limiting their ability to train and complete the experimental procedures of the study. However, to be eligible for participation, they had never been supplemented with Cr or maltodextrin or had never used anabolic steroids or beta-agonists. After baseline testing, players were ranked on muscle power output, and then matched pairs were randomly allocated in a double-blind fashion into a Cr (*N* = 9) or placebo (*N* = 10) group. Originally, there were twenty players (ten in each group), but in the second week after the beginning of the experimental period, one player left Cr group due external problems. Descriptive data of the participants are shown in Table 1.

**Table 1:** Descriptive data of the participants (*N* = 19)

	Cr Supplementation (*N* = 9)	Placebo (*N* = 10)
	Mean ± SD	Mean ± SD
Age (yrs)	16.9 ± 0.6	17.1 ± 0.4
Body weight (kg)	66.8 ± 3.2	74.2 ± 2.5 ^a^
Height (cm)	176.1 ± 5.4	178.4 ± 4.0
Body fat (%)	11.4 ± 3.3	11.7 ± 3.1
O_2max_ (mL.kg^−1^.min^−1^)	55.3 ± 1.3	51.1 ± 2.1
VT (mL.kg^−1^.min^−1^)	41.7 ± 1.8	40.7 ± 0.9
HR_max_ (bpm)	195.8 ± 1.8	197.0 ± 2.2

*O*_*2max*_ maximal oxygen uptake, *VT* ventilatory threshold, *HR*_*max*_ maximal heart rate

^a^Denotes a significant difference between the Cr and placebo groups (*P* < 0.05)

### Study design

This study used a two-group matched, double-blind, randomly assigned design. Before and after supplementation, participants completed two sessions, scheduled on different days with at least 48 h in-between session. During the first testing session, they underwent anthropometric measurements and a maximal graded exercise test using the protocol proposed by Helgerud et al. [2] to determine both their ventilatory threshold and maximal oxygen uptake. During the second testing session, they performed a 30s Wingate Anaerobic Test (WAnT) [17] to assess peak power output (PPO), mean power output (MPO), fatigue index (FI), and total work. To avoid any circadian variation, all sessions were conducted at the same time of day (±1 h). All players were familiarized with the testing procedures, having previously undertaken the tests many times. The test-retest intraclass correlation coefficients of the testing procedure variables used in this study were greater than 0.91, and the coefficients of variation ranged from 0.9 to 7.3% (unpublished data). Before the beginning of the experimental period, players were instructed to refrain from heavy exercise and avoid alcoholic or caffeinated products in the 24 h preceding the tests and to present themselves at the experimental settings in a 2 h post-absorptive state.

### Supplementation procedure

After baseline testing, soccer players were asked to consume either 0.03 g.kg.d^−1^ of creatine monohydrate (Phosphagen HP, EAS Inc., Golden, USA) or an equivalent volume of maltodextrin (Malto, NeoNutri Inc., Poços de Caldas, Brazil) for 14 days. Each supplement was measured using electronically calibrated scales and placed in identical coded airtight bags [11]. To avoid potential bias, supplements were prepared in powder form with similar texture and appearance. Players mixed the supplement powder into approximately 0.25 L of warm-to-hot water for better dissolution of Cr [9] and ingested the solution with mid-day meals. They were instructed to ingest the supplements with food because this enhances body Cr retention [19]. Cr and placebo were administered in a double-blind fashion. Both Cr and placebo supplementation regimens were initiated right after the baseline testing and ended the same day of the first testing post supplementation session. Compliance, assessed by return of empty supplement airtight bags, with the supplement was greater than 99%.

All players were asked to maintain their normal dietary behaviors throughout the study. Food diaries were given to each player to record food and fluid consumption for 4 d before the beginning of the experimental period, and players were asked to replicate this during posttraining testing. They were instructed how to report food and fluid consumption on food diaries by a co-investigator trained in clinical nutrition.

### Testing procedures

During the first session, players underwent anthropometric measurements. Body weight and height were measured according to the techniques described by Gordon et al. [20] and body fat was estimated from the measurements of seven skinfold thickness [21]. A maximal graded exercise test using the protocol suggested by Helgerud et al. [2] was then performed on a motorized treadmill (Millennium Super ATL, Imbramed, Porto Alegre, Brazil). After 10 min standard warm up consisting of running at 9 km.h^−1^, the treadmill speed was increased by 1.0 km.h^−1^ every 5 min until the point of voluntary exhaustion. All players were verbally encouraged to undertake a maximum effort. The criteria for achieving a maximal oxygen uptake required participants to meet one of the following: (a) a plateau in oxygen uptake (change of <150 mL.min^−1^ in the last three consecutive 15 s averages), (b) a respiratory exchange ratio of > 1.10, and (c) a heart rate within 10 beats.min^−1^ of the maximal level predicted by age [22]. Thus, maximal oxygen uptake was defined as the highest oxygen uptake value attained after reaching the aforementioned criteria. Ventilatory threshold (VT) was determined offline for each participant by plotting the ventilatory equivalent as a function of oxygen uptake in order to identify the point during test where this curve has its minimum value [23]. Both maximal oxygen uptake and ventilatory threshold measurements were used in this study as descriptive data (Table 1).

Oxygen uptake was measured on a breath-by-breath basis by a portable gas analysis system (K4b2, Cosmed, Rome, Italy). The system was calibrated using room air (21% O2, 0.03% CO2) and a certified gas mixture (16% O2, 5% CO2; Scott Medical Products, Plumsteadville, PA) before each test. In addition, the turbine flowmeter was calibrated with a 3 L syringe before each test.

During the second session, players performed a 30s Wingate Anaerobic Test (WAnT) [17]. Prior to testing, they were fitted for their optimal seat height on a cycle ergometer (Cybex Metabolic System, Lumex, Ronkonkoma, USA). The seat height was adjusted so that no more than 5° of knee flexion was present when the leg was extended. After a warm-up period of 5 min pedaling at 60 rpm, interspersed with 4 all-out sprints lasting 5 s, players were allowed 5 s of loadless pedaling to reach maximum cadence and were instructed to maintain maximal pedal speed throughout the 30s once the resistance was applied (0.09 kg^−1^body weight) [17, 24, 25]. Data were recorded for the 30s using a computerized WAnT program. All players were verbally encouraged to undertake a maximum effort. MPO was defined as the average of six 5 s power outputs. PPO was defined as the highest 5 s power output during the 30s test. FI was defined as ((PPO – minimum power output)/PPO) × 100 and expressed as percent of power decrement (%). Total work was defined as the summation of all six 5 s power outputs [13, 17, 24, 25].

### Statistical analysis

Descriptive data are expressed as means ± standard deviation. Data normality was evaluated with the Shapiro-Wilk *W* test for normality [26]. A two-tailed unpaired *t* test was used to compare baseline differences among the two groups’ initial muscle power output measures [27]. A series of two-factor, group (Cr supplementation and placebo) × time (pre- and post-supplementation), analysis of variance (ANOVA) with repeated measures was used to examine the pattern of change in muscle power output measures from before to after supplementation [28]. When a significant *F* value was achieved, Scheffé *post hoc* procedures were used to locate the pairwise differences between the means. Statistical significance was assumed at 5% (*P* < 0.05) a priori. All statistical analyses were performed using SPSS 18.0 for Windows (SPSS Inc., Chicago, USA). On the basis of a statistical power of 0.80, a moderately large effect size (0.25) [29], and a statistical significance of 0.05, 10 players were required for each of the cells.

## Results

The descriptive data of the participants are presented in Table 1. There were no significant differences in participant characteristics between the Cr supplementation and placebo groups (*P* > 0.05), with exception of body weight. Specifically, the soccer players in the placebo group were significantly heavier than in the Cr supplementation group (*P* < 0.05).

**Fig. 1:**
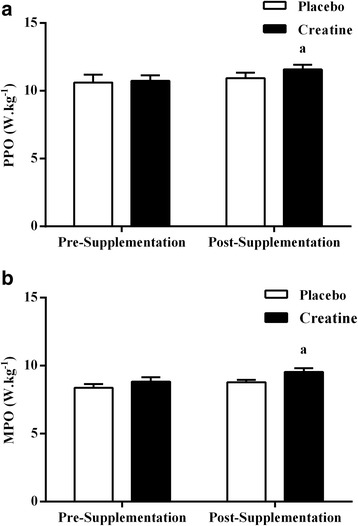
Peak power output (PPO, W.kg^−1^; **a**) and mean power output (MPO, W.kg^−1^; **b**) before and after the Cr or placebo supplementation period. ^a^ Denotes a significant difference between pre- and post-supplementation (*P* < 0.05). Values are mean ± SE

The data for the PPO (expressed in Watts per kilogram of body weight; W.kg^−1^) produced pre- and post-supplementation are presented in Fig. 1a. There were significant increases in PPO after the Cr supplementation period (8%; *P* ≤ 0.05) but not the placebo period (3%). However, there were no differences in PPO between the Cr and placebo groups pre- and post-supplementation (*P* > 0.05), with no significant interaction between group and time (*P* = 0.35). Figure 1b shows the data of MPO (expressed in Watts per kilogram of body weight; W.kg^−1^) produced pre- and post-supplementation in the Cr and placebo groups. Similarly to PPO, there were significant increases in MPO after the Cr supplementation period (8%; *P* ≤ 0.05) but not the placebo period (4%). However, there were also no significant differences in MPO pre- and post-supplementation between the Cr and placebo groups (*P* > 0.05), with no significant interaction between group and time (*P* = 0.49).

**Fig. 2:**
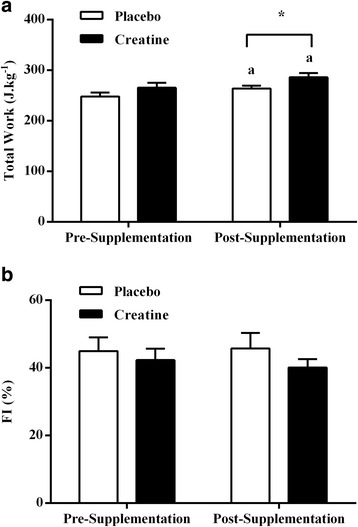
Total work (J.kg^−1^; **a**) and fatigue index (FI, %; **b**) before and after the Cr or placebo supplementation period. ^a^ Denotes a significant difference between pre- and post-supplementation (*P* < 0.05). ^*^ Denotes a significant difference between the Cr and placebo groups (*P* < 0.05). Values are mean ± SE

The data for the total work (expressed in Joules per kilogram of body weight; J.kg^−1^) produced pre- and post-supplementation are presented in Fig. 2a. There were significant increases in total work after the Cr supplementation (7%) and placebo (6%) periods (*P* < 0.05). There were also significant differences in total work between the Cr and placebo groups after (*P* < 0.05) but not before the 14 days supplementation period (*P* > 0.05), with no significant interaction between group and time (*P* = 0.88). Figure 2b shows the data of FI (expressed in percent of power decrement; %) produced pre- and post-supplementation in the Cr and placebo groups. There were no significant changes in FI after the Cr supplementation or placebo period (*P* = 0.91). There were also no significant differences in FI pre- and post-supplementation between the Cr and placebo groups (*P* > 0.05), with no significant interaction between group and time (*P* = 0.58).

## Discussion

The present study is the first to examine the effects of a low dose, short-term oral Cr monohydrate supplementation (0.03 g.kg.d^−1^ during 14 d) on muscle power output in elite youth soccer players. Few studies has been demonstrate that supplementation with Cr monohydrate in young soccer players improved soccer-specific skill performance compared with ingestion of placebo [30, 31]. Ostojic [31] in his study examined the effects of acute Cr monohydrate supplementation on soccer-specific performance in young soccer players. Twenty young male soccer players (16.6 ± 1.9 years) participated in the study and were matched and allocated to 2 randomly assigned trials: ingesting Cr monohydrate supplement (3 × 10-g doses) or placebo for 7 days. Specific dribble test times improved significantly in the Cr group (13.0 ± 1.5 vs. 10.2 ± 1.8 s; *p* < .05) after supplementation protocol. Sprint-power test times were significantly improved after Cr monohydrate supplementation (2.7 ± 0.4 vs. 2.2 ± 0.5 s; *p* < .05) as well as vertical jump height (49.2 ± 5.9 vs. 55.1 ± 6.3 cm; *p* < .05) in Cr trial. He concluded that supplementation with Cr in young soccer players improved soccer-specific skill performance compared with ingestion of placebo [31]. The major finding was that despite there being no significant difference in post-supplementation muscle power output between Cr and placebo groups (with exception of total work), the Cr supplementation resulted in significant increases in PPO, MPO, and total work. These data suggest that Cr supplementation regimens with doses as low as 2 to 3 g.d^−1^ (0.03 g.kg.d^−1^) for only 14 days beneficially affect muscle power output in youth soccer players. These data also indicate that a typical “loading phase” of Cr supplementation regimen including an increased dose of 20 to 25 g.d^−1^ (0.3 g.kg.d^−1^) for 5 to 7 days to maximize muscle total Cr content [15] might not be necessary to enhance the team sports players’ capacity to perform high intensity exercise bouts. Numerous researchers in the past [32–35] demonstrated that acute Cr supplementation has an ergogenic potential for highly trained soccer players. Cr-supplemented players showed an improved single and repeated sprint performance, increased jump ability, and enhanced endurance and agility, however, any of them aimed to analyze these effects in young soccer players nor with lower doses.

The efficacy of the low dose, short-term oral Cr supplementation protocol was demonstrated through the increases in PPO, MPO, and total work from the WAnT (8, 8, and 7%, respectively; Figs. 1 and 2). These changes were of similar magnitude to previous studies [36–38] using a “loading phase” of about 20 to 25 g.d^−1^ of Cr. For example, Birch et al. [36] reported that ingesting 4 × 5 g.d^−1^ of Cr for 5 d resulted in significant increases in PPO, MPO, and total work performed in the first two of three maximal 30s isokinetic cycling tests. Using a similar Cr supplementation protocol of 4 × 5 g.d^−1^ for 5 d and two maximal 30s isokinetic cycling tests, Casey et al. [37] found a 4% increase in the total work performed. These results, together with the present findings, confirm that the “loading phase” of the Cr supplementation is effective but unnecessary to enhance muscle power output in humans. These results also reinforce that a short-term Cr supplementation period (<14 days standard protocol) is sufficient to promote changes in muscle power output. These findings are consistent with the previous observations [39] that the majority of muscle Cr uptake appears to occur during the initial days of Cr supplementation, with a lower retention of the administered dose during subsequent supplementation days. Data from Hultman et al. [15] found that greater muscle creatine uptake during the first 14 days of low dose (0.03 g.kg.d^−1^) supplementation in comparison with the last 14 days (30% or 14 g versus 12% or 5 g).

The mechanisms behind the ergogenic effects of Cr ingestion are multifactorial and have yet to be fully elucidated [40]. It is speculated, however, that the increased capacity to generate power output after Cr ingestion might be attributed, at least in part, to increases in intramuscular total Cr concentration [7], increased ATP provision and attenuated reduction in ATP during maximal or near maximal exercise bout [8] and an increased potential for Cr acts as an cellular buffer on hydrogen ions produced during anaerobic glycolysis [41]. It is likely that this ability to generate more power output during high intensity, exercise bouts after oral Cr supplementation might allow athletes to train at higher intensities and facilitate their skeletal muscle adaptive responses to exercise training [42].

Most researchers have reported an increase in body weight after Cr ingestion [11, 18, 43, 44], although this topic has not been confirmed by some studies [12, 17]. These changes in body weight might be attributed, at least in part, to a positive nitrogen balance, which causes an increase in protein synthesis rate [41]. This possibility, however, appears to be more plausible as a result of longer periods of Cr intake combined with resistance training programs [8]. Thus, it is likely that gains in body weight are related more to greater water retention during short-term Cr supplementation than to lean-tissue accretion [15]. In the present study, however, there was no significant increase in body weight (unpublished data). Potentially, the increased total body water associated with Cr supplementation does not occur with low doses of Cr ingestion. Further research is required to examine different dosing protocols and assess total body water at multiple time points over several weeks. However, from a practical standpoint, this additional finding of absence of weight gain after a low-dose, short-term Cr supplementation may be particularly advantageous for youth athletes who weight gain is undesirable, as this may affect in sports where the categories are divided by the weight.

Previous research has reported that Cr supplementation is related to negative side effects and health risks [44]. Anecdotal reports of gastrointestinal disturbances, dehydration, and muscle cramping have been associated with Cr supplementation [45]. Controlled studies on Cr supplementation, however, have failed to support this notion [8, 11]. Consistent with this observation, no reports of gastrointestinal distress and/or medical problems/symptoms were reported by the young athletes during the Cr supplementation period. There was also no evidence of muscle cramping or muscle injury during soccer training and during testing trials (unpublished data). These results indicate that alternative Cr supplementation regimens with doses of Cr as low as 0.03 g.kg.d^−1^ for a shorter period of 2 weeks without using a prior “loading phase” are safe when used by youth athletes. This finding might be particularly useful for athletes who avoid a “loading phase” when beginning a Cr supplementation program. However, accepting that every sports and each position in that sports present different physical and physiological challenges, further researches should analyze the applicability of these short terms, low doses of Cr supplementation, focusing in the specificity of different sport and positions.

## Conclusions

In summary, the present results indicate that a low-dose, short-term oral Cr monohydrate supplementation (0.03 g.kg.d^−1^ for 14 d) beneficially affected muscle power output in youth soccer players, without any adverse side effects. Cr-supplemented soccer players showed improved PPO and MPO after the low-dose, short-term Cr supplementation (*P* < 0.05). However, placebo-supplemented soccer players also showed a small, but nonsignificant (*P* > 0.05), increase in both PPO and MPO. This small increase in PPO and MPO in placebo-supplemented soccer players, however, might explain, at least in part, the lack of significant differences between the groups following the short-term supplementation period. Therefore, we concluded that “Cr-supplemented soccer players showed improved PPO, MPO, and total work after the low-dose, short-term Cr supplementation” mainly for two reasons: 1) Cr-supplemented soccer players really showed increases in MPO, PPO, and total work after a low-dose, short-term Cr supplementation (within group differences, pre vs post analysis); and 2) placebo supplementation was not enough to cause any significant increase in both PPO and MPO in soccer players (*P* > 0.05).

The dose used in this study appears to be the lowest effective dose of Cr seen in the current literature [12], even when Cr supplementation periods are as short as 14 days. These findings of beneficial effects of a low dose, short-term Cr supplementation are of importance for applied sport scientists, nutritionists, and strength and conditioning professionals by helping them to design better nutritional interventions aimed to improve muscle power output in elite youth soccer players.
